# Comparison of Glucose Monitoring Methods during Steady-State Exercise in Women

**DOI:** 10.3390/nu4091282

**Published:** 2012-09-14

**Authors:** Stefanie J. Herrington, David L. Gee, Shireen D. Dow, Keith A. Monosky, Erika Davis, Kelly L. Pritchett

**Affiliations:** Department of Nutrition, Exercise, and Health Sciences, Central Washington University, 400 E. University Way MS 7572, Ellensburg, WA 98926, USA; Email: sjherr21@gmail.com (S.J.H.); geed@cwu.edu (D.L.G.), shireen.dow@gmail.com (S.D.D.); monoskyk@cwu.edu (K.A.M.); DavisE@cwu.edu (E.D.)

**Keywords:** continuous glucose monitoring, exercise, women, blood glucose

## Abstract

Data from Continuous Glucose Monitoring (CGM) systems may help improve overall daily glycemia; however, the accuracy of CGM during exercise remains questionable. The objective of this single group experimental study was to compare CGM-estimated values to venous plasma glucose (VPG) and capillary plasma glucose (CPG) during steady-state exercise. Twelve recreationally active females without diabetes (aged 21.8 ± 2.4 years), from Central Washington University completed the study. CGM is used by individuals with diabetes, however the purpose of this study was to first validate the use of this device during exercise for anyone. Data were collected between November 2009 and April 2010. Participants performed two identical 45-min steady-state cycling trials (~60% P_max_) on non-consecutive days. Glucose concentrations (CGM-estimated, VPG, and CPG values) were measured every 5 min. Two carbohydrate gel supplements along with 360 mL of water were consumed 15 min into exercise. A product-moment correlation was used to assess the relationship and a Bland-Altman analysis determined error between the three glucose measurement methods. It was found that the CGM system overestimated mean VPG (mean absolute difference 17.4 mg/dL (0.97 mmol/L)) and mean CPG (mean absolute difference 15.5 mg/dL (0.86 mmol/L)). Bland-Altman analysis displayed wide limits of agreement (95% confidence interval) of 44.3 mg/dL (2.46 mmol/L) (VPG compared with CGM) and 41.2 mg/dL (2.29 mmol/L) (CPG compared with CGM). Results from the current study support that data from CGM did not meet accuracy standards from the 15197 International Organization for Standardization (ISO).

## 1. Introduction

Maintaining glycemic control and avoiding hypo- and hyperglycemia during and after exercise can be challenging for physically active individuals with type 1 diabetes [1]. In addition to those performing regular physical activity, an increased number of individuals with diabetes are participating in endurance events [2]. Prolonged or strenuous exercise may increase the risk for hypoglycemia for up to 24 h after exercise; often requiring more extensive blood glucose monitoring to prevent nighttime hypoglycemia [3,4,5,6,7]. 

Continuous Glucose Monitoring (CGM) systems can provide continuous glucose concentrations to individuals with diabetes. The majority of CGM systems estimate glucose concentrations by measuring interstitial fluid glucose (IFG) and can be worn for 3 to 7 days at a time [8]. With the Dexcom (San Diego, CA) SEVEN PLUS, a small platinum wire (sensor) is inserted into the subcutaneous tissue of the abdominal region (DexCom specific) and IFG is measured continuously via a glucose oxidase reaction [8,9]. The receiver records the average glucose concentration every 5 min and stores glucose trend reports [8]. CGM-generated continuous glucose values and glucose rate of change information may be beneficial for physically active individuals with diabetes [2,5,10].

The use of CGM has been reported to improve long-term glycemic management, without increasing hypoglycemic episodes [11,12,13,14,15,16]. Some studies have used CGM to evaluate glucose profiles before and after exercise [2,17,18]. However, the reliability of individual values from CGM when blood glucose is changing rapidly remains controversial [19,20,21]. Because individuals may benefit from the convenience of fewer finger sticks, it is important to compare CGM to other blood glucose measurement methods during and after exercise. Although these devices were developed for individuals with diabetes, it is important to validate their use during exercise for those without diabetes to document “normal” responses for future comparative studies. The purpose of this study was to compare the DexCom SEVEN^®^ PLUS CGM system (IFG) to venous plasma glucose (VPG) and capillary plasma glucose (CPG) measurements during exercise and recovery in individuals without diabetes to estimate its value as a training or research tool. The study hypothesized that the difference between CGM and VPG (reference method), as well as CPG would suggest acceptable agreement between methods during moderate exercise.

## 2. Experimental Section

### 2.1. Study Population

Volunteers were recruited over a 5-month period through class announcements and flyers at Central Washington University (CWU) by the lead investigators. Enrollment criteria included: age between 18 and 44 years, non-pregnant, absence of diabetes diagnosis or hypoglycemic episodes, recreationally active individuals (≥20 min of moderate-intensity aerobic exercise, ≥three times per week) [22], and a “low risk” health status based on the American College of Sports Medicine [23]. Health status was assessed with a medical health history questionnaire. All study procedures were approved by the CWU Human Subjects Review Committee and written informed consent was given by all participants.

### 2.2. Experimental Design

The single group experimental design required participants to wear a SEVEN^®^ PLUS CGM system for 6 to 7 continuous days and perform the following three exercise sessions: peak oxygen consumption (VO_2peak_) and two identical steady-state cycling trials. Participants abstained from caffeine, alcohol, and strenuous exercise for ≥24 h prior to day 1, and ≥8 h before subsequent cycling sessions; otherwise, diet was not controlled in this study. Participants were assessed for descriptive data: age (years), height (cm), weight (kg), and body mass index (BMI; calculated as kg/m^2^). Body fat percentage was estimated by a trained investigator with Lange skinfold calipers (Cambridge, MD) using a three-site method (tricep, suprailiac, thigh) [24] and Siri equation [23]. The SEVEN^®^ PLUS sensor was inserted just under the skin of the abdominal tissue by a DexCom-trained investigator on day 1, and remained in place until completion of the study. Participants were instructed to enter 2 CPG values into the CGM device at initiation to calibrate the device, then 1 CPG value whenever the device requested for further calibration purposes. The CGM adjusts based on the CPG glucose values, but continues to display IFG.

#### VO_2peak_ Protocol (Day 1)

VO_2peak_ was estimated during an incremental exercise test to volitional exhaustion on a cycle ergometer to determine exercise intensity for submaximal trials. Gas exchange was measured using a two-way breathing valve (Hans Rudolph, Kansas City, MO) attached to a metabolic measurement system (Parvo Medics’ TrueOne^®^ 2400, UT). Heart rate (Polar electro OY, Polar, Stamford, CT) and rating of perceived exertion (RPE), using a Borg 6- to 20-point scale [25], was recorded every minute. After a 2-min warm-up, the resistance was increased by 0.5 kilopond every 2 min for the first 4 min; after which resistance increased every minute until the individual reached volitional exhaustion (VO_2peak_) [26]. Participants’ peak power outputs (P_max_) were defined as the maximum resistance achieved at volitional exhaustion [23].

### 2.3. Steady-State Cycling Sessions

Participants arrived in a fasted state (≥8 h) at 6:30 a.m. on days 2 and 4. Following baseline blood glucose collection, participants completed a 5-min warm-up. Participants then cycled at a workload corresponding to ~60% *P*_max_ for 45 min, while maintaining a cadence of 60 revolutions per minute. Fifteen min into the cycling trial, participants ingested two PowerBar (Glendale, CA) gel supplements (each containing 110 kcals, 27 g carbohydrate, 0 g protein, 0 g fat, 200 mg sodium, 20 mg potassium, and 10 g sugar) along with 360 mL of water within 5 min. 

### 2.4. Measurements during Steady-State Cycling Tests

Blood glucose was measured at baseline, every 5 min during exercise and for 15 min after exercise, using three techniques: venous blood sampling through an indwelling catheter, capillary blood draw, and interstitial fluid using CGM. During each trial, a total of 13 samples were collected from each method.

**VPG was used as the reference method in this study**. A sterile, over-the-needle catheter was used for serial blood sampling during exercise. The catheter was cannulated into an arm vein by a trained technician and its patency was maintained with a standard saline lock technique. Venous blood was drawn into heparinized vacutainers (4 mL) and immediately placed on ice. Samples were then separated with a refrigerated centrifuge for 10 min (Precision Durafuge 200R, Thermo Scientific, Waltham, MA). Glucose concentrations were measured using a glucose oxidase colorimetric assay (Pointe Scientific Inc, Canton, MI) measured at absorption of 540 nm with an UV-Visible Recording Spectrophotometer UV-160U (Shimadzu Corp, Kyoto, Japan). The average absorbance of triplicate samples was used for comparison unless outliers existed. Individual absorbance values >10% from the mean at that time point were considered outliers and excluded from the average.

CPG was measured via finger sticks with an Ascensia Contour glucometer (Bayer Healthcare LLC, Mishawaka, IN). The SEVEN^®^ PLUS estimated blood glucose via a glucose oxidase reaction, and reported an average every 5 min [8,9]. Participants were instructed to calibrate the CGM system with a CPG value every 12 h and additionally if indicated by the CGM receiver.

### 2.5. Statistical Analysis

Based on data from previous research, to achieve a power of 0.80, based on a mean glucose of 100 mg/dL and an estimated effect size of 1.0 standard deviation (~20 mg/dL), at least 10 participants were needed for this study [5,18,27]. Descriptive characteristics (mean ± standard deviation) were computed. Blood glucose data from the two submaximal trials were analyzed individually to examine agreement between blood glucose measurement devices. Absolute differences for glucose values were determined at each time point between glucose measurement methods, and mean differences calculated. Product-moment correlation coefficients were used to assess the relationship between methods. A Bland-Altman analysis was used for error analysis to determine the agreement between CGM-estimated, VPG, and CPG values [28], by computing the differences between values from each measurement method at each time point and plotting it against the average of the 2 devices. Statistical analyses were performed using Microsoft Excel (Microsoft Corporation 2007, Redmond, WA) and SPSS 16.0 (2007, Chicago, IL), with statistical significance set at *P* ≤ 0.05.

## 3. Results

### 3.1. Participant Characteristics

Twelve female participants without diabetes completed the study. Participants were 21.8 ± 2.4 years of age, normal weight (BMI 22.8 ± 2.3), 20.7% ± 4.3% body fat, and had a VO_2peak_ of 39.7 ± 4.0 mL/kg/min.

### 3.2. Blood Glucose Analysis

**Figure 1:**
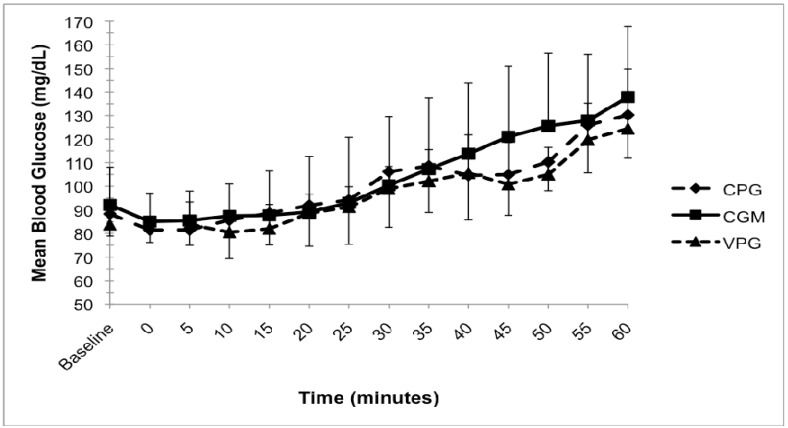
Effect of exercise and carbohydrate supplementation on glucose response during 45 min of steady-state moderate-intensity cycling and 15 min of recovery, performed by healthy females in a study to determine the comparability of three glucose monitoring methods. Average glucose values for each measurement method taken at 5 min intervals. VPG = Venous plasma glucose, CGM = Continuous glucose monitoring; CPG = Capillary plasma glucose.

Figure 1 displays the average glucose values for each device during the submaximal exercise. There was a 17.4 mg/dL and 15.5 mg/dL mean absolute difference between VPG and CGM-estimated values and between CPG and CGM-estimated values, respectively. On average, there was a tendency for CGM to overestimate VPG. In addition, mean absolute difference of 11.6 mg/dL was identified between VPG and CPG values.

**Figure 2:**
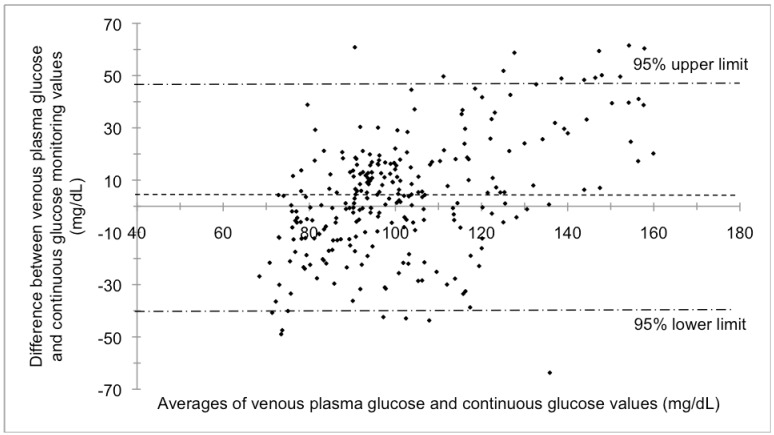
Limits of agreement between venous plasma glucose and continuous glucose monitoring-estimated values during 45 min of steady-state moderate-intensity cycling and 15 min of recovery (*r* = 0.5) performed by healthy females in a study to determine the comparability of three glucose monitoring methods. 4.4% of values outside the limits of agreement fell above the upper limit.

There was moderate positive correlation between VPG and CGM-estimated glucose values for 271 paired samples (*r* = 0.6, *P* < 0.05). Similar findings were found between CPG and CGM-estimated glucose values for 285 paired samples (*r* = 0.6, *P* < 0.05). Bland-Altman analysis suggested unsatisfactory agreement between VPG and CGM-estimated values (*r* = 0.5; mean difference 4.0 ± 22.6 mg/dL; 0.22 ± 1.25 mmol/L) (Figure 2) and between CPG and CGM-estimated values (*r* = 0.4; mean difference 3.0 ± 21.0 mg/dL; 0.17 ± 1.17 mmol/L). See Figure 2 for similar representation of CPG compared to CGM-estimated values. 

The 95% limits of agreement were defined as 2 standard deviations from the glucose mean difference (1.96 times standard deviation) [28]. The limits of agreement were ±44.3 mg/dL (2.46 mmol/L) for VPG *vs.* CGM-estimated values and ±41.2 mg/dL (2.29 mmol/L) for CPG *vs.* CGM-estimated values. The limits of agreement between each of these methods tended to increase as average glucose increased. When the steady-state cycling trials were broken into stages, the increase in mean difference and limits of agreement were identified as follows for VPG compared to CGM values: baseline (mean difference ± 2 standard deviations) 1.7 ± 25.1 mg/dL (0.09 ± 1.40 mmol/L), exercise only (5–15 min) 0.8 ± 26.5 mg/dL (0.04 ± 1.47 mmol/L), exercise plus carbohydrate (20–45 min) 2.1 ± 47.8 mg/dL (0.12 ± 2.65 mmol/L), and post-exercise (50–60 min) 11.4 ± 53.7 mg/dL (0.63 ± 2.98 mmol/L). A similar trend was found when comparing CPG to CGM values: baseline 1.3 ± 21.3 mg/dL (0.07 ± 1.18 mmol/L), exercise only (5–15 min) 1.7 ± 22.9 mg/dL (0.09 ± 1.27 mmol/L), exercise plus carbohydrate (20–45 min) 2.0 ± 48.1 mg/dL (0.11 ± 2.67 mmol/L), and post-exercise (50–60 min) 6.8 ± 45.1 mg/dL (0.38 ± 2.50 mmol/L).

Results from the current study did not meet the International Organization for Standardization 15197 (ISO) standards for accuracy [29,30]. Using glucometers, 95% of values ≥75 mg/dL (4.2 mmol/L) should be within ±20% of the blood glucose concentration, and within ±15 mg/dL (0.83 mmol/L) for values <75 mg/dL (4.2 mmol/L), to be considered acceptable [29,30]. Only 69.4% and 72.7% of CGM-estimated values ≥75 mg/dL (4.2 mmol/L)) were within the ISO 15197 standards when compared to VPG and CPG, respectively. For CGM-estimated values <75 mg/dL (4.2 mmol/L) only 27.6% and 51.7% met these standards when compared to VPG and CPG, respectively.

## 4. Discussion

The purpose of the current study was to examine the acceptability of using a CGM system to estimate blood glucose during exercise with a carbohydrate feeding. CGM systems were developed to help improve daily glycemic control and prevent detrimental episodes of hypo- or hyperglycemia. The validation of CGM devices in the diabetes population, as an adjunct for improving daily glycemic control is well documented [11,12,13,14,15,16]; however limited research regarding their acceptability for use during exercise and in those without diabetes remains questionable [2,5,10,17,20,27]. An athlete’s blood glucose during exercise has the potential to increase/decrease rapidly and can be difficult to monitor. The interference of self-monitoring of blood glucose, or finger stick, testing during intense training or competition may disturb an athletes’ performance [6,20]. For individuals with diabetes, hypo- and hyperglycemia are common after strenuous endurance events, lasting up to 24 h [3,7]. Therefore, CGM may be beneficial for athletes because CGM provides continuous readings and glucose rate of change information that warns individuals about rapidly increasing or decreasing glucose profiles. Despite the limited studies examining the point-accuracy of CGM systems (available in the US) during exercise, CGM has been shown to be useful during exercise by helping athletes understand personal glycemic profiles during and after activity [2,5,10,17].

Similar to other research findings [5,18,27], the current study found a large potential variable difference between CGM-estimated and VPG, as well as CPG values during exercise. In contrast, some studies report an overall acceptable agreement between measures (CGM, venous, and capillary) [18,27]. Results from the current study revealed absolute mean differences similar to other findings using the DexCom SEVEN^®^ in non-exercise studies (12.8% to 16.3% mean absolute relative differences) [31,32].

The correlation between CGM values and blood glucose in the current study was lower than found in previous studies (*r* = 0.8 to 0.9) [5,27]. Similar results have been reported when comparing CGM values to either CPG or VPG values [5,33]. Discrepancy in this difference might be related to a smaller blood glucose range in the current study compared to previous research with individuals with diabetes. In contrast to the current study, other researchers have reported satisfactory agreement between VPG and CGM-estimated values during exercise, using Bland-Altman analysis [18,27], despite having larger mean differences.

Under moderate-intensity cycling, resting, and recovery conditions in the current study, CGM on average, had a tendency to overestimate mean VPG. Similarly, MacDonald *et al.* [18] reported a tendency of overestimation by another CGM system, during moderate-intensity exercise. Furthermore, the consistency of the differences between the methods increased in concordance with average glucose [18]. However, Fayolle *et al.* [27] reported an average overestimation with the GlucoDay^®^ (A. Menarini Diagnostics, Florence, Italy) during low-intensity exercise (mean difference 16.4 ± 4.1 mg/dL, *P* < 0.0001); with a tendency to underestimate VPG during high-intensity exercise (−25.9 ± 11.0 mg/dL). Some literature suggests that IFG may decrease more rapidly than blood glucose during exercise because of the rapid uptake of glucose by the muscles [17]. However, in the current study, average CGM values tended to overestimate VPG during exercise and recovery. In addition, IFG via CGM was measured in adipose tissue of the abdominal region, not in the exercising muscle. Nevertheless, it has been proposed that IFG differs insignificantly between body compartments [34]. This can present as a confounding variable because of the potential differences between glucose uptake in interstitial fluid surrounding muscles and adipose tissue. Due to the nature of this study, it is likely that a physiological lag-time between these compartments might have contributed to differences found in the study.

Since CPG is the standard measure for individuals with diabetes during normal daily activities, we examined whether CGM is acceptable compared to CPG. Several others support this in their research by using CPG; although, most often using VPG as their standard during exercise and non-exercise states [5,33,35,36]. In contrast to at least one study [37], the current study reported that CGM overestimated CPG on average. Few studies during exercise have examined the accuracy between CGM and CPG values. Of these studies, Iscoe *et al.* [5] identified a strong correlation (*r* = 0.89, *P* < 0.001) between these two methods during exercise. However, Wilhelm *et al.* [35] reported no significant differences between CPG and CGM values during non-exercise states.

Although Bland-Altman plots identified some agreement between blood glucose methods, these results did not meet the ISO standards [29,30]. When analyzed by stage, the limits of agreement between CGM and VPG values and between CGM and CPG values increased as the exercise progressed. One exception was found between CPG and CGM values during recovery. Therefore, under the current study conditions, the CGM system appeared to be more acceptable at the beginning of exercise, possibly because fewer variables were imposed on the participant, and the rate of glucose change was negligible, whereas blood glucose increased rapidly later during exercise [32]. To our knowledge, current research has not examined the addition of carbohydrate feeding during exercise on CGM acceptability.

This study was not without limitations. Because glucometers may have acceptable deviation of ±20%, the accuracy of relying on a single CPG measure at each time point, as done in the current study, may not be an accurate comparison in such a research study. Averages of triplicate measurements might yield more accurate results for CPG.

It has been proposed that CGM may not be as accurate or effective in individuals without diabetes because of narrow daily glucose ranges [5,18]. McGowan *et al.* [33] reported weaker correlations in participants with diabetes with the tightest daily blood glucose ranges, suggesting that narrower glucose profiles may result in greater variance of CGM [33]. However, other studies using CGM in healthy populations without diabetes report similar variance between CGM-estimated and VPG values, as well as with CPG compared to individuals with diabetes [18,21]. Mean differences in the current study were smaller than reported by MacDonald *et al.* [18], however both suggested similar overestimations of blood glucose by CGM. Although the range of blood glucose concentrations in individuals without diabetes is more limited, the present results shed light on differences in accuracy in methods for measuring blood glucose, regardless of whether individuals have diabetes. These results can be used as “reference values” in individuals without diabetes for future comparative studies. Although MacDonald *et al.* [18] reported similar variance to the present study, based on results from McGowan *et al.* [33], we suggest CGM devices may be suitable for individuals with diabetes to use during exercise. Future comparative studies involving CGM devices, exercise and individuals with diabetes would help to confirm this notion.

## 5. Conclusions

In conclusion, based on the results of the current study, CGM was not found to consistently provide acceptably accurate glucose values when compared to VPG or CPG during moderate-intensity cycling and recovery. These results provide information on the accuracy of CGM during exercise, but cannot be directly translated to non-exercise conditions. Therefore, CGM should be used in conjunction with standard glucometers during and after exercise to improve accuracy and confirm extreme values before taking corrective action. Further research is warranted to examine the acceptability of CGM during exercise in individuals with and without diabetes. 

## References

[1] Hornsby W.G., Chetlin R.D. (2005). Management of Competitive Athletes. Diabetes Spectr..

[2] Cauza E., Hanusch-Enserer U., Strasser B., Ludvik B., Kostner K., Dunky A., Haber P. (2005). Continuous Glucose Monitoring in Diabetic Long Distance Runners. Int. J. Sports Med..

[3] Briscoe V.J., Tate D.B., Davis S.N. (2007). Type 1 Diabetes: Exercise and Hypoglycemia. Appl. Physiol. Nutr. Metab..

[4] Gulve E.A. (2008). Exercise and Glycemic Control in Diabetes: Benefits, Challenges, and Adjustments to Pharmacotherapy. Phys. Ther..

[5] Iscoe K.E., Campbell J.E., Jamnik V., Perkins B.A., Riddell M.C. (2006). Efficacy of Continuous Real-Time Blood Glucose Monitoring during and after Prolonged High-Intensity Cycling Exercise: Spinning with a Continuous Glucose Monitoring System. Diabetes Technol. Ther..

[6] MacKnight J.M., Mistry D.J., Pastors J.G., Holmes V., Rynders C.A. (2009). The Daily Management of Athletes with Diabetes. Clin. Sports Med..

[7] Sandoval D.A., Guy D.L., Richardson M.A., Ertl A.C., Davis S.N. (2004). Effects of Low and Moderate Antecedent Exercise on Counterregulatory Responses to Subsequent Hypoglycemia in Type 1 Diabetes. Diabetes.

[8] (2008). SEVEN^®^ PLUS Continuous Glucose Monitoring System Users Guide.

[9] Burge M.R., Mitchell S., Sawyer A., Schade D.S. (2008). Continuous Glucose Monitoring: The Future of Diabetes Management. Diabetes Spectr..

[10] Manders R.J.F., van Dijk J.M., van Loon L.J.C. (2010). Low-Intensity Exercise Reduces the Prevalence of Hyperglycemia in Type 2 Diabetes. Med. Sci. Sports Exerc..

[11] Bailey T.S., Zisser H.C., Garg S.K. (2007). Reduction in Hemoglobin A1c with Real-Time Continuous Glucose Monitoring: Results from a 12-week Observational Study. Diabetes Technol. Ther..

[12] Deiss D., Bolinder J., Riveline J., Battelino T., Bosi E., Tubiana-Rufi N., Kerr D., Phillip M. (2006). Improved Glycemic Control in Poorly Controlled Patients with Type 1 Diabetes Using Real-Time Continuous Glucose Monitoring. Diabetes Care.

[13] Deiss D., Hartmann R., Hoeffe J., Kordonouori O. (2004). Assessment of Glycemic Control by Continuous Glucose Monitoring System in 50 Children with Type 1 Diabetes Starting on Insulin Pump Therapy. Pediatr Diabetes.

[14] Garg S., Jovanovic L. (2006). Relationship of Fasting and Hourly Blood Glucose Levels to HbA1c Values. Diabetes Care.

[15] Garg S., Zisser H., Schwartz S., Bailey T., Kaplan R., Ellis S., Jovanovic L. (2006). Improvement in Glycemic Excursions with a Transcutaneous, Real-Time Continuous Glucose Sensor. Diabetes Care.

[16] Ludvigsson J., Hanas R. (2003). Continuous Subcutaneous Glucose Monitoring Improved Metabolic Control in Pediatric Patients with Type 1 Diabetes: A Controlled Crossover Study. Pediatrics.

[17] Cauza E., Hanusch-Enserer U., Strasser B., Kostner S.K., Dunky A., Haber P. (2005). Strength and Endurance Training Lead to Different Post Exercise Glucose Profiles in Diabetic Participants Using a Continuous Subcutaneous Glucose Monitoring System. Eur. J. Clin. Invest..

[18] MacDonald A.L., Philp A., Harrison M., Bone A.J., Watt P.W. (2006). Monitoring Exercise-Induced Changes in Glycemic Control in Type 2 Diabetes. Med. Sci. Sports Exerc..

[19] Boyne M.S., Silver D.M., Kaplan J., Saudek C.D. (2003). Timing of Changes in Interstitial and Venous Blood Glucose Measured with a Continuous Subcutaneous Glucose Sensor. Diabetes.

[20] Chassin L.J., Wilinska M.E., Hovorka R. (2007). Intense Exercise in Type 1 Diabetes: Exploring the Role of Continuous Glucose Monitoring. J. Diabetes Sci. Technol..

[21] Monsod T.P., Flanagan D.E., Rife F., Saenz R., Caprio S., Sherwin R.S., Tamborlane W.V. (2002). Do Sensor Glucose Levels Accurately Predict Plasma Glucose Concentrations during Hypoglycemia and Hyperinsulinemia?. Diabetes Care.

[22] Brown C.N., Mynark R. (2007). Balance Deficits in Recreational Athletes within Chronic Ankle Instability. J. Athl. Train..

[23] American College of Sports Medicine (2009). Guidelines for Exercise Testing and Prescription.

[24] Pollock M.L., Schmidt D.H., Jackson A.S. (1980). Measurement of Cardiorespiratory Fitness and Body Composition in the Clinical Setting. Compr. Ther..

[25] Borg G. (1982). Psychophysical Bases of Perceived Exertion. Med. Sci. Sports Exerc..

[26] Maud P.J., Foster C. (2006). Physiological Assessment of Human Fitness.

[27] Fayolle C., Brun J.F., Bringer J., Mercier J., Renard E. (2006). Accuracy of Continuous Subcutaneous Glucose Monitoring with the GlucoDay in Type 1 Diabetic Patients Treated by Subcutaneous Insulin Infusion during Exercise of Low *vs.* High Intensity. Diabetes Metab..

[28] Bland M.J., Altman D.G. (1986). Statistical Methods for Assessing Agreement between Two Methods of Clinical Measurement. Lancet.

[29] (2008). Performance Metrics for Continuous Interstitial Glucose Monitoring.

[30] Young J.K., Ellison J.M., Marshall R. (2008). Performance Evaluation of a New Blood Glucose Monitor that Requires No Coding: The OneTouch^®^ Vita™ System. J. Diabetes Sci. Technol..

[31] Kamath A., Mahalingam B.J. (2009). Analysis of Time Lags and Other Sources of Error of the DexCom SEVEN Continuous Glucose Monitor. Diabetes Technol. Ther..

[32] Weinstein R.L., Schwartz S.L., Brazg R.L., Bugler J.R., Peyser T.A., McGarraugh G.V. (2007). Accuracy of the 5-Day FreeStyle Navigator Continuous Glucose Monitoring System. Diabetes Care.

[33] McGowan K., Thomas W., Moran A. (2002). Spurious Reporting of Nocturnal Hypoglycemia by CGMS in Patients with Tightly Controlled Type 1 Diabetes. Diabetes Care.

[34] Cengiz E., Tamborlane W.V. (2009). A Tale of Two Compartments: Interstitial Blood Glucose Monitoring. Diabetes Techol. Ther..

[35] Wilhelm B., Forst S., Weber M.M., Larbig M., Pfützner A., Forst T. (2006). Evaluation of CGMS® during Rapid Blood Glucose Changes in Patients with Type 1 Diabetes. Diabetes Technol. Ther..

[36] Zavalkoff S.R., Polychronakos C. (2002). Evaluation of Conventional Blood Glucose Monitoring as an Indicator of Integrated Glucose Values Using a Continuous Subcutaneous Sensor. Diabetes Care.

[37] Bode B., Gross K., Rikalo N., Schwartz S., Wahl T., Page C., Gross T., Mastrototaro J. (2004). Alarms Based on Real—Time Sensor Glucose Values Alert Patients to Hypo—and Hyperglycemia: The Guardian Continuous Monitoring System. Diabetes Technol. Ther..

